# Nutrient optimization for indigenous microbial consortia of a Bhagyam oil field: MEOR studies

**DOI:** 10.3389/fmicb.2023.1026720

**Published:** 2023-03-16

**Authors:** Neha Sharma, Meeta Lavania, Vatsala Koul, Dhruva Prasad, Nitish Koduru, Amitabh Pandey, Rahul Raj, M. Suresh Kumar, Banwari Lal

**Affiliations:** ^1^Microbial Biotechnology, Environmental and Industrial Biotechnology Division, The Energy and Resources Institute (TERI), New Delhi, India; ^2^Cairn Oil and Gas, Vedanta Limited, ASF Center, Gurugram, India

**Keywords:** microbial enhanced oil recovery, GC-MS, RSM, pathogenicity assay, core flood test

## Abstract

The microbial enhanced oil recovery (MEOR) method is an eco-friendly and economical alternative technology. The technology involves a variety of uncertainties, and its success depends on controlling microbial growth and metabolism. This study is one of a kind that showed successful tertiary recovery of crude oil through indigenous microbial consortia. In this study, a medium was optimized to allow ideal microbial growth under reservoir conditions through RSM. Once the nutrient recipe was optimized, the microbial metabolites were estimated through gas chromatography. The maximum amount of methane gas (0.468 mM) was produced in the TERIW174 sample. The sequencing data set showed the presence of *Methanothermobacter* sp. and *Petrotoga* sp. In addition, these established consortia were analyzed for their toxicity, and they appeared to be safe for the environment. Furthermore, a core flood study showed efficient recovery that was ~25 and 34% in TERIW70 and TERIW174 samples, respectively. Thus, both the isolated consortia appeared to be suitable for the field trials.

## Introduction

Global energy demand and consumption are predicted to increase, with fast-growing economies accounting for more than half of the growth in energy demand (Sarkar et al., 2016; Nikolova and Gutierrez, 2020). Among the world's total energy sources, oil is one the most widely utilized fuel and accounts for more than half of the total energy consumption (BP., 2017). However, 70% of the total petroleum hydrocarbon reserves are highly viscous, making them incompatible with processing. Asphaltene, resins, and petroleum alkanes usually form a dynamically stable system in crude oil. Petroleum alkanes usually act as solvents, asphaltene act as micelles, and resins act as stabilizers (Shibulal et al., 2014). Nevertheless, the asphaltene component is the heaviest component of crude oil as it is viscous, flocculating, and contains a significant amount of heteroatoms [sulfur, nitrogen, and oxygen (S, N, O)] that adversely affect the production, transportation, and processing of petroleum (Zou, 2017). As a result, wells and pipelines become partially clogged, making oil production more difficult and expensive (Zou et al., 2020).

Due to the depletion of light oil resources and sustained high oil prices, the primary focus is shifting toward the utilization of heavy oil reserves. World reserves of heavy hydrocarbons are estimated to be ~70%. However, the viscosity of heavy crude oil is significantly high (Li et al., 2022). Therefore, conventional recovery processes are expensive and have low sweep efficiency. Often, heating consumes a significant amount of energy, and diluents such as water have some logistical challenges (Geetha et al., 2018).

The microbial mechanism involves the synthesis of secondary metabolites such as biosurfactants that can reduce the interfacial tension between the aqueous phase and residual saturation (Nikolova and Gutierrez, 2020). A report hypothesized that bacterial metabolites such as organic acid, biogas, biosurfactants, and biopolymers may significantly improve oil recovery by reducing viscosity. Thus, crude oil's recovery efficiency is improved (Tabatabaee and Assadi, 2013). Microbes have evolved specifically for high-temperature petroleum reservoirs at great depth because they are found in high-temperature reservoirs (Gomes et al., 2016). Hence, being indigenous (native), they only require nutrients to grow and are easily able to perform such activities. Several thermophilic reservoir bacteria can use crude oil as an exclusive carbon and energy source to produce metabolites (surfactant, emulsifier) (Daryasafar et al., 2016).

Bhagyam is an onshore field in the Rajasthan state of western India and is part of Mangala-Bhagyam-Aishwariya (MBA) development in the Barmer basin. The main producing unit is Fatehgarh multi-storied fluvial sandstone. Reservoir quality is excellent with permeability in the range of 1 to 10 Darcy and porosity in the range of 25–30%. The crude oil is moderately viscous (15 to 500 cP). The field has been developed with downdip water injection, and after successful evaluation of the long-term polymer injectivity test, it is currently under full-field polymer flood implementation. The novelty of this study is to explore the efficiency of microbial-enhanced oil recovery in Bhagyam fields, which has not yet been investigated in the reservoir systems. The overall cost of the MEOR trial will be economical as compared to other recovery processes. In the present study, the indigenous microbial species was grown in optimized media conditions, and optimization was performed through the RSM methodology. The anaerobic bacteria were characterized through molecular tools and were morphologically identified. The microbial species that was capable of growing under reservoir conditions was taken up for core flood assay and acute oral toxicity to determine the ecotoxicity of the consortia.

## Materials and methods

### Sampling site

**Figure 1:**
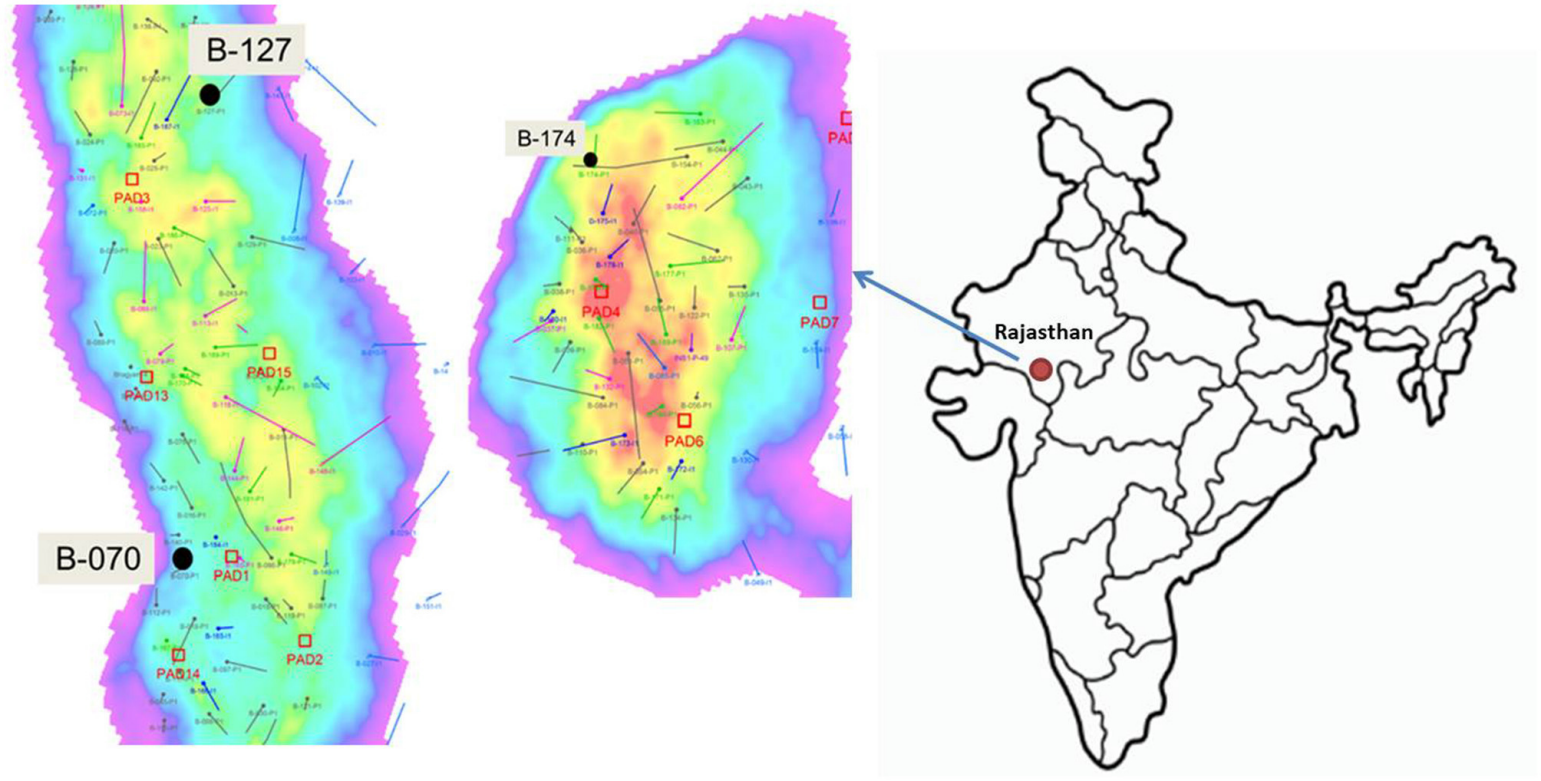
Stratigraphy of Bhagyam oil fields and the neighboring area.

Bhagyam oil field consists of 15 oil well pads with an area of 43,320 sqm and STOIIP (stock tank oil initially in place) over 500 MMbbls, and is located in Barmer, Jaisalmer, and Rajasthan, with a longitude of 25.7521°N and latitude of 71.3967°E (Figure 1). In the first phase of collection, three samples of water (formation water) and crude oil were collected from the Bhagyam oil field on 10 May 2019. The climate is generally dry with a maximum average temperature of ~51°C and an average minimum temperature of 0°C. The average rainfall was ~277 mm. The well details are as follows: Well 70, Well 127, and Well 174 with bottom hole temperatures of 48°C, 48°C, and 52°C, respectively.

### Physicochemical estimation of formation water and oil

Physicochemical analysis, including pH, conductivity (mS/cm), total dissolved solids (g/l), salinity (PSU), heavy metals (arsenic, cadmium, chromium, copper, zinc, silver, and nickel) (mg/l), and elements (carbon, hydrogen, nitrogen, and sulfur) (ppm) was performed. The analysis was performed according to the standard guideline of the API (American Petroleum Institute) and APHA (American Public Health Association).

Total petroleum hydrocarbons (TPH) were analyzed in crude oil, as reported by Lavania et al. TPH was determined by the gravimetric method followed by gas chromatography analysis. A glass column was filled with a slurry of 60–120 mesh silica gel (activated at 70°C for 16 h) in n-hexane. After the silica gel settled, the hexane was drained until its level reached the top of the silica gel. The concentrated sample (0.5 g) dissolved in n-hexane was placed on the column. The column was then developed successively, with n-hexane and toluene to obtain saturated (n-hexane) and aromatic (toluene) fractions. These fractions were collected in separate pre-weighed dishes and were evaporated in a fume hood for gravimetric and gas chromatography analyses (Lavania et al., 2015).

### Optimization of the nutrient recipe for establishing ideal microbial growth

Response surface methodology (RSM) using Box–Behnken design was applied to optimize the three important medium components, namely, 0–3 gL^−1^ ammonium chloride (X1) serves as a nitrogen source and 1.0–3.5 gL^−1^ sodium bicarbonate (X2) and 1.5–8.5 gL^−1^ molasses (X3) act as a carbon source, favoring maximum metabolite production through a microbial consortia of Well 70 and Well 174, namely, TERIW70 and TERIW174 (institute name followed by the well no.) using Design Expert. Other components of the media included phosphates (K_2_HPO_4_) and sulfates (MgSO_4_.7H_2_O), which were required in minute amounts. To investigate the most appropriate combination of these three components, 20 sets of experiments (also known as run orders) were generated (Supplementary Table S1). Contour plots were generated to understand the interaction of different variables and were used to find the optimum concentration of the medium components affecting the response. Experiments were performed in 67 mL serum bottles containing 30 mL of different components of the anaerobic media and were incubated at 50°C with 10% (v/v) bacterial inoculum (cell count 10^5^ cells mL^−1^). The growth was estimated through gases and VFA production after the incubation period (Sharma et al., 2020).

### Growth and screening of indigenous consortia in optimum media conditions

An enrichment technique was employed to isolate the indigenous microbial consortia from the formation water samples. The optimized anaerobic Baltch media (NH_4_Cl, 1.5 g/l; Molasses, 8.5 g/l; NaHCO_3_, 2.25 g/l; K_2_HPO_4_, 0.1g/l; MgSO_4_.7H_2_O, 0.2 g/l; and L-cysteine, 0.5 g/l) was prepared through sparging of nitrogen gas that removes the dissolved oxygen from the water. Approximately 30 ml of media was dispensed in a 67 ml serum bottle specially designed for anaerobic cultures. The media bottles were sealed with an aluminum crimp and autoclaved for 15 min at 121°C. Approximately 10% v/v formation water sample was inoculated in the cool media and incubated at 50°C for 10 days. After 10 days of incubation, the bottles were analyzed for various metabolites such as headspace gases and volatile fatty acids through gas chromatography (GC 7890A Agilent Ltd., USA), as described in a previous study (Sharma et al., 2017).

### Viscosity reduction experiment

This experiment was conducted in a 110 ml anaerobic serum bottle containing 70 ml of media with 10% oil (7 ml). The enriched culture was served as inoculum (TERIW70 and TERIW174) and added into the sterilized media bottles at a rate of 10% (v/v). The viscosity measurement was conducted by using a cone-plate viscometer (Brookfield-Programmable R/S + CPS + Rheometer) on the 30th day of incubation at reservoir temperature (50°C). For the viscosity measurement, 7 ml of extracted oil fraction was placed and sheared between a stationary flat spindle (C75-1) at a pre-set shear stress (1 Pascal). The torque exerted on the spindle by the oil fraction was proportional to the shear stress. This viscosity (shear stress/shear rate) was measured from a pre-calibrated system. The viscosity data were recorded at 70°C, where the sample exhibited signs of a Newtonian fluid. The constant temperature during the viscosity measurement was maintained by using a heated circulating water bath (Heto CBN 18-50/HMT 200) with an accuracy of ±0.1°C. The viscosity measurements for all samples were carried out at 10 different time points for a 60 s interval. This was repeated three times, and the average values of the three measured viscosity data were noted (Lavania et al., 2015).

### Molecular identification and morphological characterization of consortia

Based on the metabolite generation, two bacterial consortia, TERIW70 and TERIW174, were included in the genome profiling. Whole genomic DNA was extracted and amplified using PCR primers targeting the hypervariable region of 16s rRNA V3-V4 (forward primer: 5′ TCGTCGGCAGCGTCAGATGTGTATAAGAGACAGCCTACGGGNGGCWGCAG and reverse primer: 5′ GTCTCGTGGGCTCGGAGATGTGTATAAGAGACAGGACTACHVGGGTATCTAATC). The PCR product was purified using the QIAGEN gel elution kit and sequenced with the illumina Miseq platform (Illumina, USA). Furthermore, a quality check of the FASTQ files was performed through FastQC (v0.11.9), and the sequencing data files were analyzed using the DADA2 package (version 1.14.0), which included quality filtering, trimming of barcode/adaptors, dereplication, learn error rates, chimera removal, and merging of paired reads. The SILVA version 138 16S rRNA gene reference database was used to assign bacterial taxonomic classification. Singleton reads were removed for statistical analysis. Compositions of microbiota communities were summarized by proportion at different taxonomy levels, including genus, family, order, class, and phylum ranks. The phylogenetic tree was constructed using the neighbor-joining method in MEGA X (version 10.1.8) package. The tree topologies were estimated with 1,000 bootstrap data sets. All significant sequence data have been deposited in the NCBI GenBank database (Sharma et al., 2020).

The morphology of consortia was determined through scanning electron microscopy. The dried sample was coated with carbon tape and sputter coated with a thin layer of gold. The images were examined on a Zeiss EVO MA 10.

### Toxicity determination of TERIW70 and TERIW174

Pathogenicity testing of the TERIW70 and TERIW174 microbial consortia was conducted at the National Toxicology Center (APT Testing and Research Pvt. Ltd.) in Pune, as per the guidelines of the EPA 712-C-96-315 OPPTS 885.3050. A total of 32 mice (16 males and 16 females) were assigned to the dose group: controls and trials. The test substance was administered once to mice by gavage. The mice fasted for 3–4 h and 2 h after administration of the test substance. Water was allowed to be ingested freely. At the end of the observation period, all animals were carefully examined for the presence of anaerobic bacteria. The body weight and organ weight were recorded on test days 1, 7, 14, and 21. All animals were observed for mortality throughout the observation period (Sharma et al., 2017).

### Core flood studies

**Figure 2:**
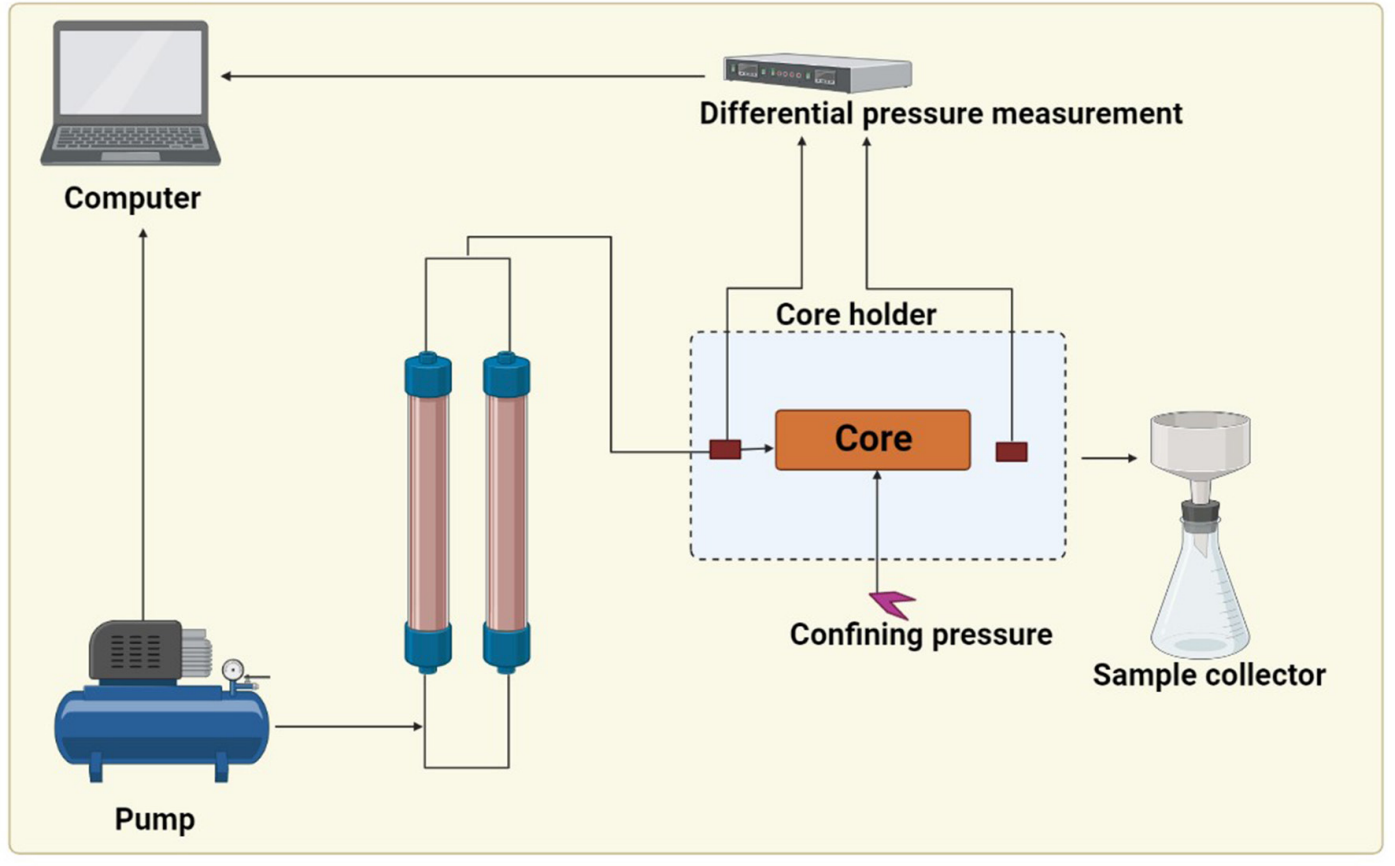
Schematic of core flood apparatus.

The core flood experiment was conducted at simulated oil reservoir conditions using oil field–injected water and crude oil under similar temperature conditions to the oil reservoir. This study was carried out at IIT (ISM), Dhanbad. The core flooding instrument consists of a core holder, displacement pump, calibrated collector, differential pressure measurement system, slug container, and an oven (Figure 2). The core holder was placed into the oven to maintain the reservoir temperature, and the pump was used to inject the fluid at a constant rate, which was stored in a stainless-steel container. The injection fluid was heated with the help of a heating jacket before entering into the core holder. An overburden pressure of 500 psi was applied with the help of a hydraulic pump by the injection of hydraulic oil between the rubber sleeve encasing the core and the core holder's internal surface. To establish the irreducible water saturation (Swi), dead crude oil was injected at a rate of 0.5 ml/min until only oil is produced at the outlet, and no more water was produced, indicating that irreducible water saturation has been reached; then cores were kept in a sealed vessel filled with the crude oil in an oven for 1 week at a temperature of 50°C. The core was remounted in the core holder, and the fluid was injected in secondary mode and tertiary mode at a flow rate of 0.5 ml/min. After the injection of microbes (TERIW70 and TERIW174) which were developed anaerobically in the optimized media at a rate of 10%, the system was aged for 1 month at a temperature of 50 °C. All the experiments were conducted at a temperature of 50 °C and an overburden pressure of 500 psi. Effluents were collected throughout the water flooding experiment using a compact collector, and the pressure drop was monitored with the help of a differential pressure gauge installed across the core holder. Effluent collected was used to determine the oil recovery.

## Results

This study focused primarily on the feasibility of MEOR technology in a “huff and puff” mode to improve injectivity/productivity and oil recovery. Bhagyam field production started with water flood, and currently, the field is mainly on polymer flood (Koduru et al., 2021). As a result of long-term exploitation, chemicals, stimulating fluids, polymers, and acidizing fluids have been injected into the oil reservoir, which impact the composition of the formation water and the types and number of microbes found in the production wells.

### Components of formation water

The concentration of hydrogen ions, total dissolved solids, and chloride ions was determined. The concentration of elements and heavy metals in all the formation water samples was determined by the APHA guidelines (Table 1).

**Table 1:** Physicochemical characterization of formation water collected from Bhagyam fields.

**Parameter**		**Sample name**		
	**Test methods**	**Well 70**	**Well 127**	**Well 174**
*pH*		8.085	7.83	7.445
*TDS (g/l)*		6.45	6.76	5.87
*Conductivity (mS/cm)*		13.35	13.52	11.77
*Salinity (PSU)*		7.35	7.7	6.67
**Elements (ppm)**				
*Carbon*	APHA/IS: 1350	0.292	0.193	0.197
*Hydrogen*	APHA/IS: 1350	8.81	10.21	10.08
*Nitrogen*	APHA/IS: 1350	0.219	0.496	0.242
*Sulfur*	APHA/IS: 1350	0.333	0.205	0.176
**Heavy metals (mg/l)**				
*Arsenic (As)*	IS3025 PT 37:1988	ND	ND	ND
*Cadmium (Cd)*	APHA3100(B)	ND	ND	ND
*Chromium (Cr)*	APHA3500(B)	ND	ND	ND
*Coppe r(Cu)*	APHA3111(B)	ND	ND	ND
*Zinc (Zn)*	APHA3100(B)	ND	ND	ND
*Silver (Ag)*	APHA3113(B)	ND	ND	ND
*Nickel (Ni)*	APHA3111(B)	ND	ND	ND

ND, not detected.

### Estimation of crude oil fraction through silica gel

Crude oil fractions after the gravimetric analysis showed that the aliphatic content was ~50% in all the samples, whereas the aromatic content was ~27% in Well 127 and Well 174, and 20% in the case of Well 70. Non-hydrocarbon compounds such as nitrogen, sulfur, and oxygen (NSO) and other metal-containing compounds were found in a range of 16–18% in the crude oil samples (Table 2).

**Table 2:** Compositional analysis of oil collected from Bhagyam fields.

**S. No**.	**Sample name**	**Compositional analysis of oil (%)**		
		**Aliphatic (%)**	**Aromatic (%)**	**Others & NSO (%)**
1.	Well 70	59.6	20.6	19.8
2.	Well 127	56	27.4	16.6
3.	Well 174	54.2	27.8	18

**Figure 3:**
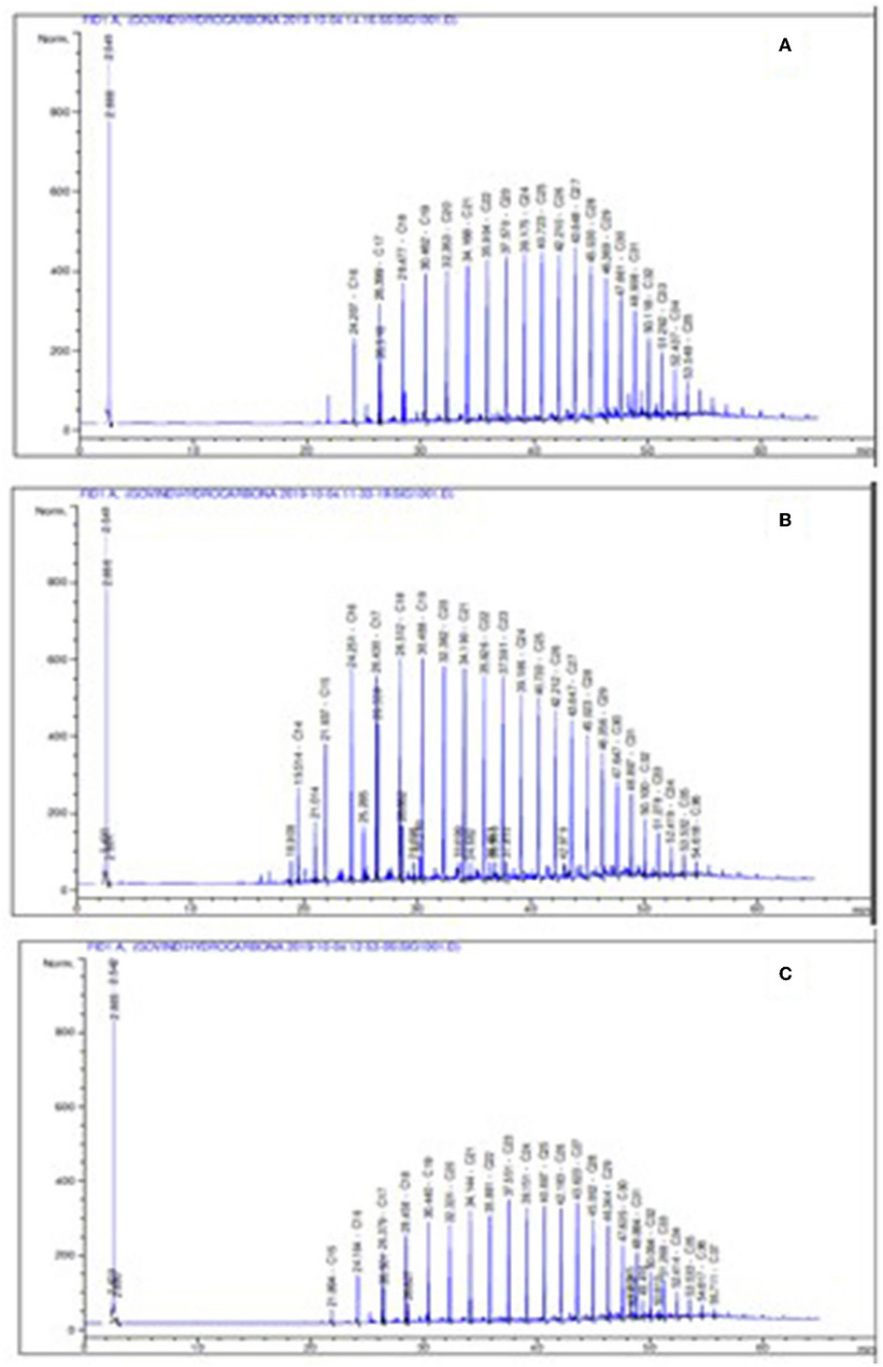
Representation of the gas chromatogram of an aliphatic fraction of crude oil of wells, where **(A)** Well 70, **(B)** Well 127, and **(C)** Well 174.

The aliphatic chain of crude oil of Well 70 shows the presence of C16–C35 carbon chains (Figure 3A), whereas crude oil of Well 127 shows the presence of C14–C36 carbon chains in the gas chromatogram (Figure 3B). The crude oil of Well 174 shows the presence of C15–C37 carbon chains in the gas chromatogram (Figure 3C).

**Figure 4:**
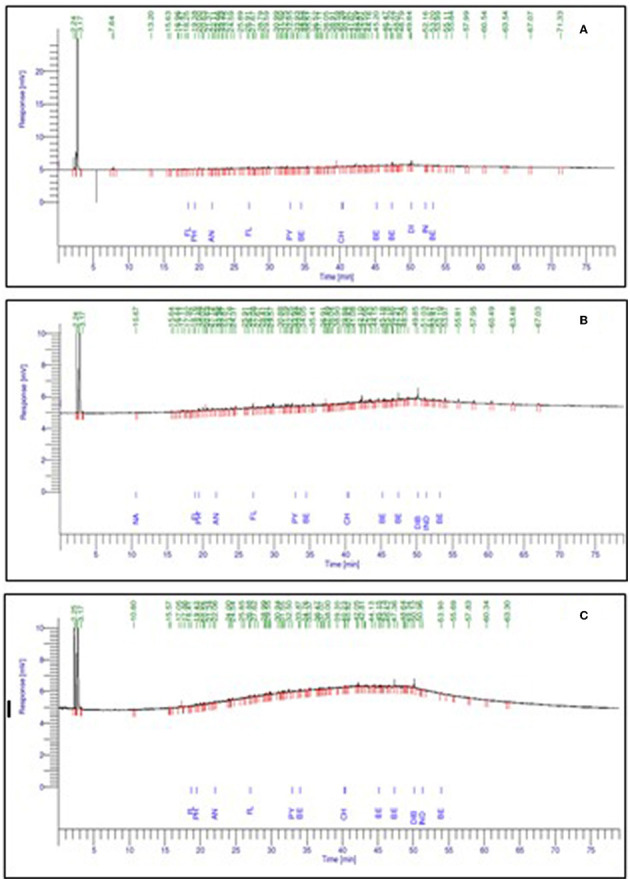
Representation of a gas chromatogram of the aromatic fraction of crude oil of wells, where **(A)** Well 70, **(B)** Well 127, and **(C)** Well 174.

The aromatic components of crude oil of Well 70 include fluorene, phenanthrene, anthracene, fluoranthene, pyrene, benzanthracene, chrysene, benzofluoranthene, benzopyrene, dibenzoanthracene, indenopyrene, and benzoperylene (Figure 4A). Crude oil of Well 127 shows the presence of naphthalene, fluorene, phenanthrene, anthracene, fluoranthene, pyrene, benzanthracene, chrysene, benzofluoranthene, benzopyrene, dibenzoanthracene, indenopyrene, and benzoperylene (Figure 4B). Crude oil of Well 174 shows the presence of fluorene, phenanthrene, anthracene, fluoranthene, pyrene, benzanthracene, chrysene, benzofluoranthene, benzopyrene, dibenzoanthracene, indenopyrene, and benzoperylene, as shown in Figure 4C.

### Enrichment of indigenous consortia of Bhagyam fields

**Figure 5:**
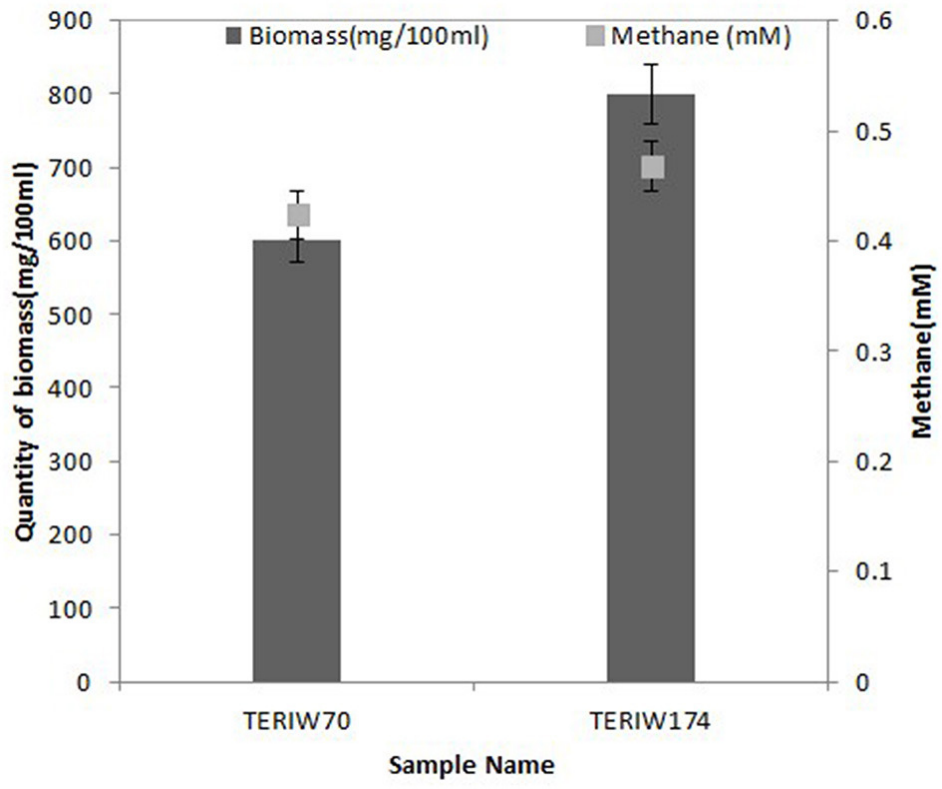
Graph representation of microbial growth in terms of biomass and methane production.

The indigenous consortia were inoculated in an optimized media, which was incubated at 50°C for 10 to 20 days. The metabolites were estimated through the defined protocol, as mentioned in the previous section. The maximum biomass obtained in inoculated cultures was ~600 mg/100 ml in formation water of Well 70 in M_2_X media. The maximum biomass of Well 174 was 800 mg/100 ml in M_2_X media. The maximum amount of methane was 0.464 mM and 0.468 mM in Well 70 and Well 174, respectively (Figure 5).

The viscosity of oil (Well 70) was gradually reduced with TERIW70 and TERIW174 consortia, representing an ~22 and 24% reduction in the dynamic viscosity (measuring unit was mPas), which indicates that the secondary metabolites, *viz*. volatile fatty acids and gases, were capable of reducing the interfacial tension between oil and water, therefore enhancing the microbial-based recovery in the field trials.

### Optimized nutrient recipe for maximum metabolite production

The RSM methodology was used to explore the interaction between the culture parameters and metabolite production by TERIW70 and TERIW174, thus increasing the economic and technological feasibility of the process. RSM (response surface methodology) is an amalgamation of different statistical techniques that include designing of experiments, building of models, and evaluating the effects of factors on responses (Rathi et al., 2015).

**Figure 6:**
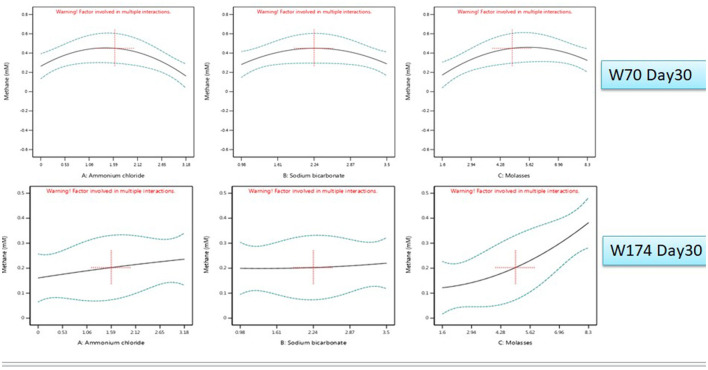
All factor interaction for methane and VFA production (day 30) using Design Expert software.

**Figure 7:**
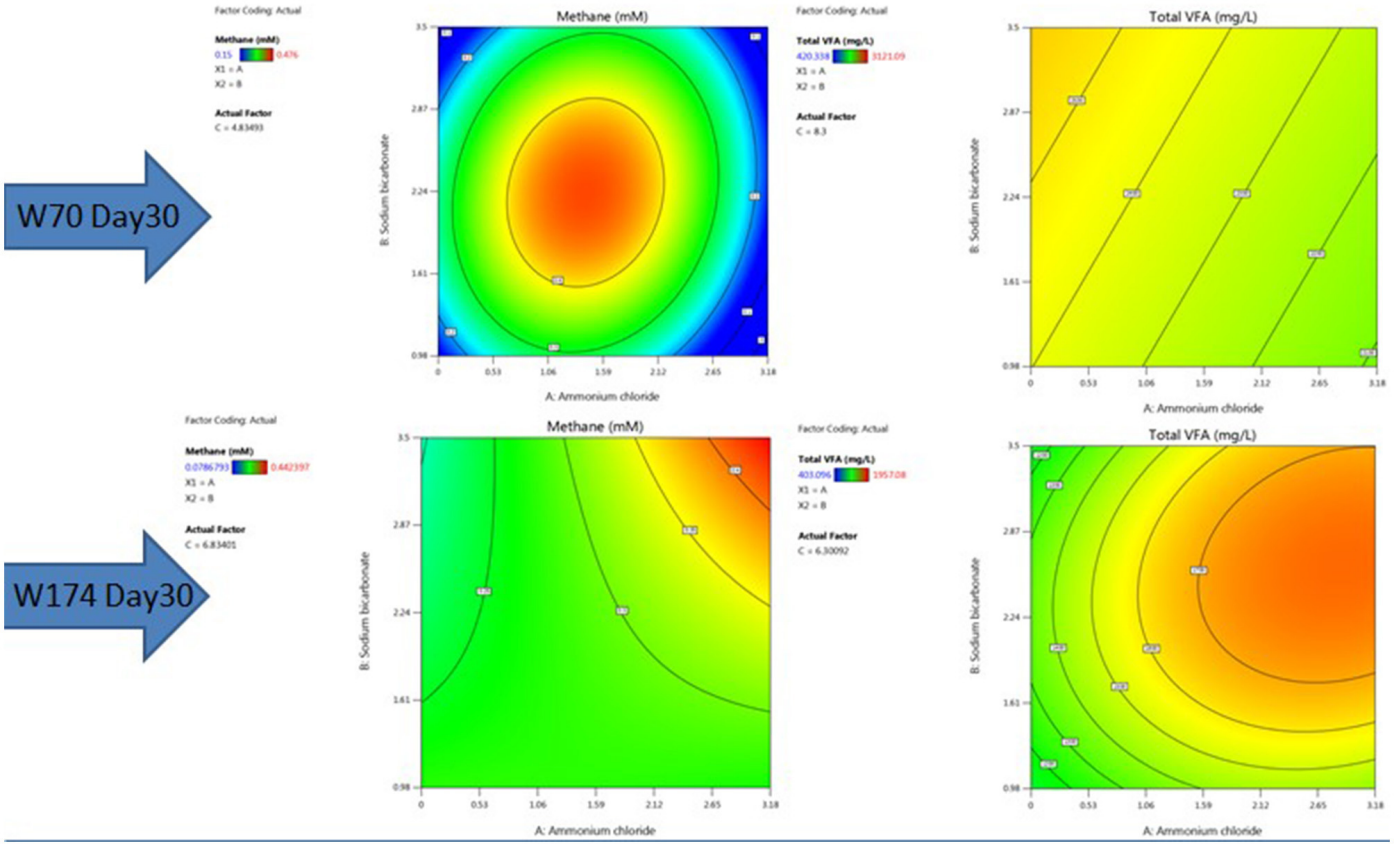
RSM contour plots of the interactive effect of ammonium chloride, sodium bicarbonate, and molasses after 30 days with TERIW70 and TERIW174.

The nutrient media optimization was performed in 67 mL serum bottles containing 30 mL of Baltch medium (M2X) with 10% (v/v) bacterial inoculum (cell count 10^5^ cells mL^−1^). The results are presented in Figures 6, 7A, B. The highest production of methane on day 30 in sample W70 was observed in Run7 (0.48 mM/bottle) and for sample W174 in Run1 (0.44 mM/bottle). Run7 consisted of 1.5 g/l of ammonium chloride, 5 g/l of molasses, and 2.25 g/l

of sodium bicarbonate, whereas Run1 consisted of 8.5 g/l of molasses and the rest of the components were the same.

For sample W70, the highest concentration of total VFAs for day 10, day 20, and day 30 (3121.09 mg/L) were found in Run1. For sample W174, the highest production of total VFAs on day 30 was observed in Run19 (1957.08 mg/L). Molasses was established as an important component supporting enhanced methane production in both W70 and W174. However, Run1 media components can perform well in terms of VFA and gases. Thus, further enrichments were carried out with the components of Run1.

### Identification and morphological characterization of the consortia

**Figure 8:**
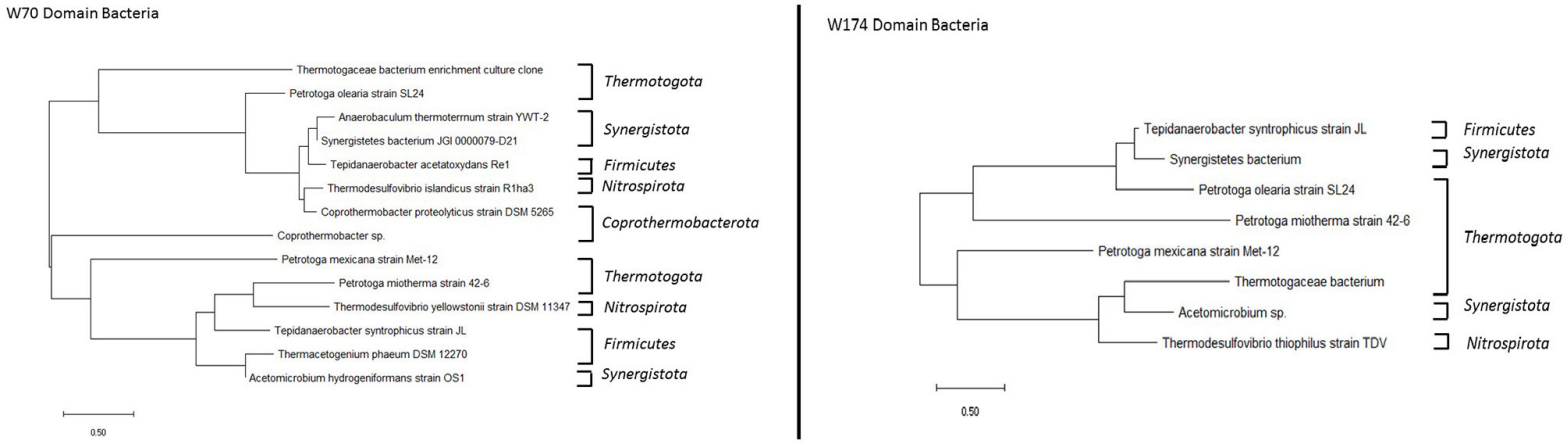
Phylogenetic tree constructed using the neighbor-joining method for consortia TERIW70 and TERIW174.

The consortia obtained from both wells included methanogenic bacteria *Methanothermobacter*. Higher bacterial diversity was observed in the sample TERIW70 (Figure 8). The most abundant species in TERIW70, which is also present in TERIW174, was *Acetomicrobium* sp., an anaerobic thermophile which converts glucose to acetate, CO_2_, and H_2_ during the process of methanogenesis. The most abundant species in TERIW174 is *Petrotoga* sp., which is a strict anaerobic, thermophilic, and methane-producing bacteria usually isolated from petroleum reservoirs (Rathi et al., 2015). *Thermodesulfovibrio* was also present in both wells, which is also an anaerobic, sulfate-reducing bacteria usually isolated from methanogenic sludge. Cheng et al. (2011) reported the presence of the genus *Methanothermobacter* in high-temperature oil reservoirs (Cheng et al., 2011).

**Figure 9:**
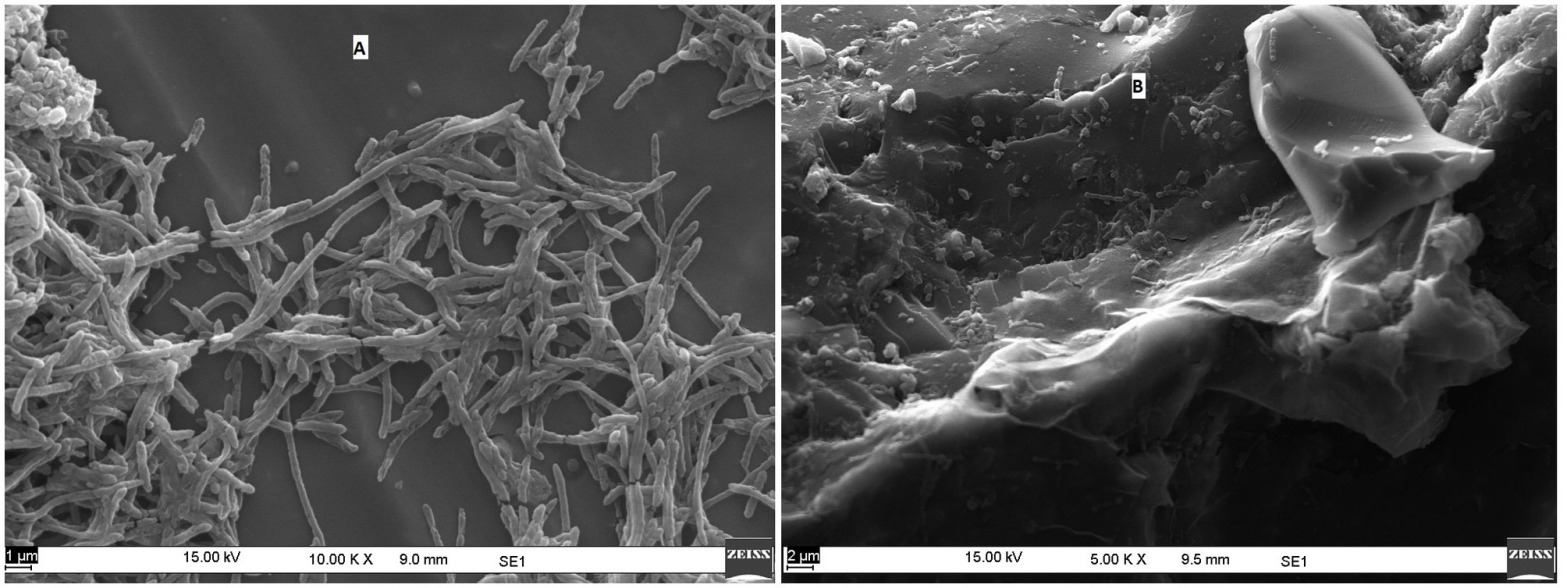
Scanning electron micrograph of **(A)** TERIW70 and **(B)** TERIW174.

The morphology of TERIW70 appeared to be slightly curved rods (0.3 μm in width and 2.2 to 5.9 μm in length) that occurred singly or in pairs. A flagellum was not observed in the micrograph (Figures 9A, B). TERIW174 dominantly showed *Petrotoga* sp., which appeared in the high-salinity oil reservoirs as rod-shaped structures.

### Pathogenicity Test of TERIW70 and TERIW174

After the end of the incubation period of 21 days, all the mice appeared normal and showed no clinical signs of intoxication after dosing in the toxicity study. For the course of the 21-day observation period, the mice receiving treatment did not have any negative effects on their body or organ weight increase. Both the samples (TERIW70 and TERIW174) did not induce any gross pathological alterations, and no live anaerobic bacteria were found in the organs of the mice. Furthermore, no mortality was observed after 21 days, and all the mice were alive. Samples were found to be safe mice by the oral route at a dose level of 1.0 ml, containing 1.0 X 10^6^ CFU.

### Core flood assay

**Figure 10:**
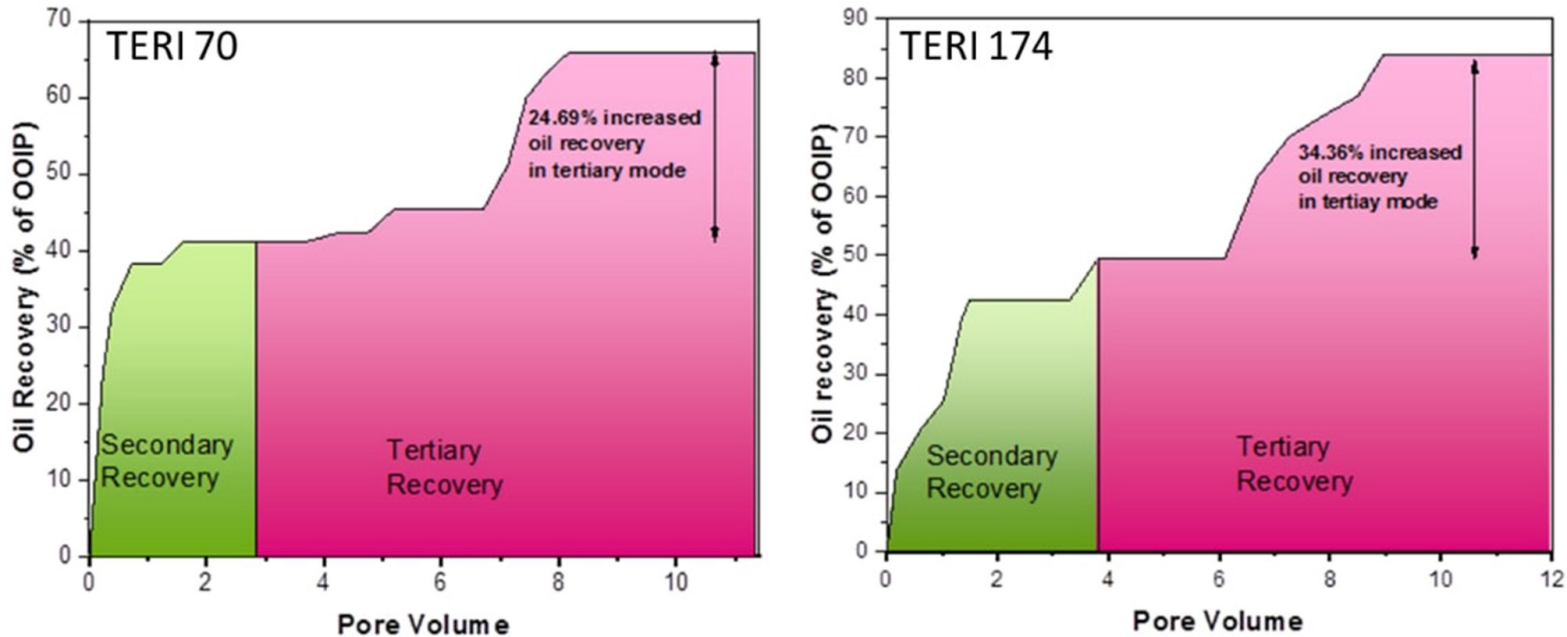
Enhanced recovery of oil by microbial injection after water flooding.

**Figure 11:**
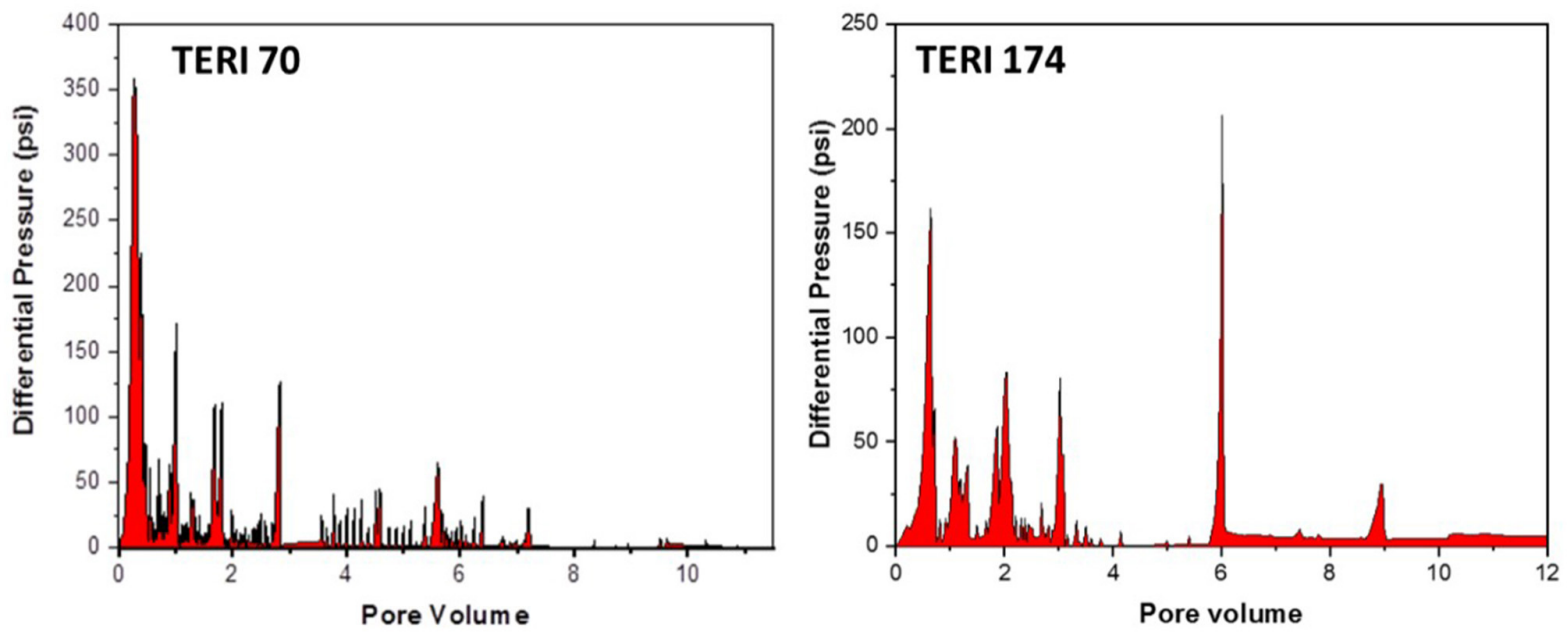
Variation of differential pressure drop with pore volume injection during microbial flooding in TERIW70 and TERIW174.

The petrophysical properties of cores used in the flooding studies are presented in Table 3. The absolute permeability, i.e., the water permeability in the current experiment, was calculated to be 216 md for TERIW70 and 527 md for the TERIW174 system. Based on the experiments performed and subsequent material balance, the oil recovery in secondary mode and tertiary mode with injection of microbial slugs was calculated. The enhanced recovery of oil by consortium TERIW70 was ~25% of the original oil in place (OOIP) after the water flooding and ~34% for TERIW174, as shown in Figure 10. Furthermore, the relationship between the differential pressure and pore volume was also estimated during microbial flooding. It can be observed from the differential pressure behavior, that, initially during water flooding, the pressure was significantly high to maintain the mobilization of oil. The crude oil provided for the experiment was observed to be thick and highly paraffinic in nature. After water flooding during the pre-flush slug, no pressure drop was observed, as the remaining residual oil was immobile. In addition, with the introduction of microbes, the viscosity of the oil and the interfacial tension between the oil and water decreases, which facilitates the mobilization of trapped oil (Sharma et al., 2020). Thus, after the injection of microbes in core flood assay, the differential pressure exhibits an abrupt change, owing to the pressure required for the mobilization of residual oil due to the non-Newtonian fluid behavior of the oil. Furthermore, due to the interactions of microbes, the IFT (interfacial tension) and viscosity decreased and the pressure drop was observed to be stable in both the consortia (Figure 11).

**Table 3:** Properties of the core sample and recovery data for consortia TERIW70 and TERIW174.

**Core properties**	**TERIW70**	**TERIW174**
Length (cm)	7.6	7.8
Diameter (cm)	3.8	3.75
Bulk volume (cm^3^)	86.192	86.1483
Dry weight of core (g)	171.12	169.19
Wet weight of core (g)	193.37	186.24
Difference	22.25	17.05
Pore volume (Ml) (P.V)	22.25	17.05
Porosity of core sample	0.2581	0.1979
Porosity of core sample (%)	25.814	19.791
Water permeability (md)	216	527
Initial water saturation (Swi In %)	23.59	14.663
Original oil in place (OOIP) (ml)	17	14.55
Secondary recovery (%)	44.117	49.484
Pre-flush recovery (%)	4.11	0
Microbial recovery (%)	14.7	27.49
Chase water recovery (%)	5.88	6.87
**Total tertiary recovery (%)**	**24.69**	**34.36**

## Discussion

Microorganisms are present in oil reservoirs around the world, where they degrade oil and lead to changes in oil quality. Unfortunately, our knowledge about processes in deep oil reservoirs is limited due to the lack of undisturbed samples. In this study, the distribution of microorganisms in Bhagyam fields was identified to be *Methanothermobacter* sp. and *Petrotoga* sp. found in TERIW70 and TERIW174. Thermotoga and *Firmicutes* are the most common thermophilic bacteria isolated from reservoirs with high temperatures. The genera *Thermotoga, Thermosipho, Geotoga*, and *Petrotoga* of the phylum Thermotogae have been isolated from diverse oil-rich habitats (Grabowski et al., 2005).

Numerous environmental factors such as temperature and salinity affect oil recovery throughout the MEOR process. Temperature determines the type of microorganisms which can inhabit the reservoir (Pannekens et al., 2019). A previous investigation on microbial communities in oil reservoirs also reported that the bacterial community was significantly influenced by reservoir environments (Gao et al., 2019). Thermophilic genera of bacteria including *Bacillus, Thermus, Thermoanaerobacter, Thermococcus*, and *Thermotoga* were isolated from high-temperature reservoirs in China, California, and the North Sea, and would therefore be ideal candidates for MEOR (Kaster et al., 2009; Lin et al., 2014). Three thermophilic hydrocarbonoclastic species of *Bacillus, Geobacillus*, and *Petrobacter* have been reported to withstand 55°C and degrade hydrocarbons under strict anaerobic conditions, suggesting their compatibility with MEOR (Shibulal et al., 2014).

The indigenous microbial consortia TERIW70 and TERIW174 were capable of synthesizing methane and biomass that help in pressuring the crude oil and in selective plugging of low permeable zones of the core. Methane (CH_4_) and carbon dioxide (CO_2_), both produced by microorganisms, have been shown to enhance oil recovery by reducing the viscosity and restoring well pressure (Gudina et al., 2013). Kobayashi et al. also demonstrated the ability of indigenous microorganisms in the reservoir to produce methane for reservoir re-pressurization (Kobayashi et al., 2012).

Using the RSM methodology, nutrient recipes were optimized to increase secondary metabolites (gases and volatile fatty acids) at minimal concentrations of medium components, thus reducing overall process costs for MEOR. At the moment, we are focused on reducing the cost of the process so that it can be economically viable. As the conditions were already optimized for the MEOR process, the nutrient recipe was optimized to enhance microbial growth within a minimum period. Consequently, the optimization study reduces the cost of the process by reducing the concentration of expensive components (Hong and Haiyun, 2010). A report showed 19.48% residual oil recovery of Mexican heavy oil in a core flood experiment using a mixed culture of extremophiles A7, indigenous to the oil field. In the MEOR process, the mixed culture A7 produced surface-active agents (surface tension reduction of 27 mN m^−1^), solvents (ethanol, 1738 mg L^−1^), acids (693 mg L^−1^), and gases and also degraded heavy hydrocarbon fractions in an extreme environment (Castorena-Cortes et al., 2012).

The application of genetically engineered microorganisms for MEOR was demonstrated by Sun et al. who created a bacterial strain, GW3-3.0, from *Enterobacter cloacae* and thermophilic *Geobacillus* sp., designed to produce polymers at higher temperatures. In assessing the strain's suitability for MEOR, GW3-3.0 was used for a core flood experiment, with promising results (Sun et al., 2011). Another report showed that a bacterial consortium growing at 91°C and above (optimally at 96°C) was capable of enhancing the oil recovery in both sand pack (26.7%) and core flood studies (10.1%) (Arora et al., 2014).

A previous report showed that *Thermoanaerobacter* sp., indigenous to the high-temperature reservoir of Gujarat, India, has the tendency to alter the permeability and sweep efficiency of high-permeability zones, facilitating the displacement of oil. In core flood studies, it showed an effective reduction in permeability at residual oil saturation; from 28.3 to 11.3 mD and a 19.2% incremental oil recovery in a core flood assay (Table 4) (Sharma et al., 2018). *Thermophilic thermus SP3* reduces the crude oil viscosity and paraffin content, which leads to the decomposition of heavy fractions, increases the light crude oil content, and further improves physical and chemical properties, thus enhancing oil recovery by 12.59% under core flooding studies (Hao and Wang, 2009).

**Table 4:** Percentage of oil recovery previously reported.

**Study**	**Consortia used**	**Oil recovery (%)**	**References**
A mixed culture A7 produced secondary metabolites.	*Thermoanaerobacter* sp.	19.48	Castorena-Cortes et al., 2012
Genetically engineered microorganisms produced polymer at high temperature.	*Enterobacter cloacae* strain	10.1	Sun et al., 2011
Indigenous sp. *Thermoanaerobacter* sp alters the sweep efficiency and permeability.	*Thermoanaerobacter* sp., *Thermoanaerobacter brockii, Thermoanaerobacter italicus, Thermoanaerobacter mathranii, Thermoanaerobacter thermocopriae*.	19.2	Sharma et al., 2018

## Conclusion and future prospects

The knowledge of utilizing microbial systems for improving the recovery of heavy oils is growing; however, microbial-based metabolites that are non-toxic and biodegradable could be promising to improve recovery and stimulate the well. Hence, the TERIW70 and TERIW174 consortia were extracted from Bhagyam fields and tested in core flood experiments under reservoir conditions, showing 25% and 34% total tertiary recovery.

The performance of the MEOR process depends on numerous environmental parameters found in the reservoir, which facilitate the growth of and metabolite formation by the microorganisms. The success rate of the MEOR process is hard to predict because of the various factors. As each reservoir has different conditions, the MEOR process must be designed in such a way that it will work effectively under field trials. Optimization of the nutrient recipe will help to minimize the cost associated with field implementation. Therefore, MEOR technology can be utilized for further recovery of dead oil wells or heavy crude oil.

## Data availability statement

The datasets presented in this study can be found in online repositories. The names of the repository/repositories and accession number(s) can be found in the article/Supplementary material.

## Ethics statement

The animal study was reviewed and approved by Pathogenicity was conducted in APT Testing and Research Pvt Ltd. (Formerly known as National Toxicology Center). The author(s) are not required to state the ethical considerations of their study in the manuscript, including cases where the study was exempt from ethical approval procedures.

## Author contributions

NS and ML: conceived and designed the experiments. VK, BL, ML, and DP: provided all the resources for performing experiments. NS: performed the experiments and analyzed the data. All authors contributed to the article and approved the submitted version.
