# Supplementary material for: Nutrient optimization for indigenous microbial consortia of a Bhagyam oil field: MEOR studies
Source: Front Microbiol. 2023 Mar 16;14:1026720. doi: 10.3389/fmicb.2023.1026720 (PMC10060980; doi:10.3389/fmicb.2023.1026720)
Supplement: Supplementary file 1 [file Data_Sheet_1.docx]

**Supplementary data**

**Nutrient optimization for Indigenous microbial consortia of a Bhagyam oil field: MEOR studies**

**Neha Sharma^a^, Meeta Lavania^a*^, Vatsala Koul^a^, Dhruva Prasad^b^, Nitish Koduru^b^, Amitabh Pandey^b^, , Rahul Raj^b^ , M Suresh Kumar^b^, Banwari Lal^a^**

^a^Microbial Biotechnology, Environmental and Industrial Biotechnology Division, The Energy and Resources Institute (TERI), New Delhi, India

^b^Cairn Oil & Gas, Vedanta Limited, ASF Center, Tower A, 362-363, Jwala Mill Road, Phase IV, Udyog Vihar, Sector 18, Gurugram, India

*** Correspondence: Dr Meeta Lavnia

[meetal@teri.res.in](mailto:meetal@teri.res.in)

**Experiment:** Optimization of nutrient recipe for maximum metabolites production.

**Methodology**

In response surface methodology (RSM), the minimum and the maximum value of three components were taken as the hold value. To determine the most suitable combination of three components, twenty (20) sets of experiments or run orders were generated as depicted in supplementary Table S1.

**Table S1.** Box Behnken design for three variables and experimentally determined actual data by RSM for enhanced production of bacterial metabolites to support the MEOR activity.

| **Run Order** | **(Components in g/l)** | | | **Methane (mM)** | | **VFA(mg/l)** | |
| --- | --- | --- | --- | --- | --- | --- | --- |
|  | **Ammonium Chloride** | **Sodium bicarbonate** | **Molasses** | **W70** | **W174** | **W70** | **W174** |
| 1 | 1.5 | 2.25 | 8.5 | 0.289805 | 0.442397341 | 3121.09238 | 1775.243716 |
| 2 | 0.5 | 1.75 | 3 | 0.365048 | 0.258051848 | 2602.731723 | 1513.694024 |
| 3 | 0.5 | 3 | 7 | 0.277274 | 0.125179007 | 572.6134846 | 625.0878838 |
| 4 | 0.5 | 0.5 | 3 | 0.244307 | 0.239095085 | 1597.291981 | 1245.614019 |
| 5 | 1.5 | 3 | 5 | 0.210973 | 0.173112916 | 907.1357459 | 979.4370243 |
| 6 | 2.5 | 1.75 | 3 | 0.154705 | 0.204290423 | 1461.321402 | 1466.61265 |
| 7 | 1.5 | 2.25 | 5 | 0.475579 | 0.078679322 | 420.5813508 | 403.0957594 |
| 8 | 1.5 | 1.75 | 1.6 | 0.150024 | 0.274081244 | 1448.259447 | 1309.81847 |
| 9 | 3.18 | 1.75 | 5 | 0.231098 | 0.229007455 | 1616.828267 | 1578.545951 |
| 10 | 0.5 | 1.75 | 7 | 0.217379 | 0.138378134 | 1442.854359 | 1123.145716 |
| 11 | 0.5 | 1.75 | 7 | 0.277376 | 0.270215593 | 420.3384881 | 1915.963899 |
| 12 | 0.5 | 1.75 | 7 | 0.283679 | 0.196416199 | 1331.750549 | 1022.254472 |
| 13 | 0 | 2.25 | 5 | 0.150388 | 0.141990532 | 968.0965365 | 865.1958458 |
| 14 | 2.5 | 1.5 | 7 | 0.286996 | 0.326935161 | 2035.351881 | 979.4370243 |
| 15 | 0.5 | 1.75 | 5 | 0.289805 | 0.442397341 | 3121.09238 | 1775.243716 |
| 16 | 0.5 | 1.75 | 5 | 0.365048 | 0.258051848 | 2602.731723 | 1513.694024 |
| 17 | 0.5 | 1.75 | 5 | 0.277274 | 0.125179007 | 572.6134846 | 625.0878838 |
| 18 | 2.5 | 1.5 | 3 | 0.244307 | 0.239095085 | 1597.291981 | 1245.614019 |
| 19 | 2.5 | 3 | 7 | 0.210973 | 0.173112916 | 907.1357459 | 1957.08063 |
| 20 | 0.5 | 1.75 | 5 | 0.475579 | 0.204290423 | 1461.321402 | 1466.61265 |

All the experiments were set for 30 days in triplicate sets. The parameters analyzed were gas and VFAs production. The data was analyzed using multiple regressions and a second order polynomial model fitted for predicted optimum levels was expressed in Equation 1.

$Y=\beta o+\sum\beta n Xn+\sum\beta nn {Xn}^{2}+\sum\beta nm Xn Xm$ Eq.1

Where, Y was the predicted response, $\beta o$ was the intercept coefficient; $\beta n$ was linear coefficient, $\beta nn wa$s quadratic coefficient and $\beta nm$ was interaction coefficient. The regression and graphical analysis was determined through Design Expert software. The interactive effects of significant variables were further represented in form of contour plots.

**Results**

The experiments were performed in random order to acquire the maximum metabolites (VFA and gases) produced as actual response. Highest production of methane in sample W70 was observed in Run7 with 0.48 mM/bottle and for sample W174 in Run1 (0.44mM/bottle). For sample W70, highest concentration of total VFAs was 3121.09 mg/L found in Run1. For sample W174, highest production of total VFAs was observed in Run19 (1957.08 mg/L).

Therefore, Run1 appeared to be the most optimum for both the metabolites (VFA and methane gas). Thus, further enrichment was performed with Run 1 media components.
